# Soluble Rhesus Lymphocryptovirus gp350 Protects against Infection and Reduces Viral Loads in Animals that Become Infected with Virus after Challenge

**DOI:** 10.1371/journal.ppat.1002308

**Published:** 2011-10-20

**Authors:** Junji Sashihara, Yo Hoshino, J. Jason Bowman, Tammy Krogmann, Peter D. Burbelo, V. McNeil Coffield, Kurt Kamrud, Jeffrey I. Cohen

**Affiliations:** 1 Medical Virology Section, Laboratory of Infectious Diseases, National Institute of Allergy and Infectious Diseases, National Institutes of Health, Bethesda, Maryland, United States of America; 2 Neurobiology and Pain Therapeutics Section, Laboratory of Sensory Biology, National Institute of Dental and Craniofacial Research, National Institutes of Health, Bethesda, Maryland, United States of America; 3 AlphaVax, Inc., Research Triangle Park, North Carolina, United States of America; University of Texas Health Science Center San Antonio, United States of America

## Abstract

Epstein-Barr virus (EBV) is a human lymphocryptovirus that is associated with several malignancies. Elevated EBV DNA in the blood is observed in transplant recipients prior to, and at the time of post-transplant lymphoproliferative disease; thus, a vaccine that either prevents EBV infection or lowers the viral load might reduce certain EBV malignancies. Two major approaches have been suggested for an EBV vaccine- immunization with either EBV glycoprotein 350 (gp350) or EBV latency proteins (e.g. EBV nuclear antigens [EBNAs]). No comparative trials, however, have been performed. Rhesus lymphocryptovirus (LCV) encodes a homolog for each gene in EBV and infection of monkeys reproduces the clinical, immunologic, and virologic features of both acute and latent EBV infection. We vaccinated rhesus monkeys at 0, 4 and 12 weeks with (a) soluble rhesus LCV gp350, (b) virus-like replicon particles (VRPs) expressing rhesus LCV gp350, (c) VRPs expressing rhesus LCV gp350, EBNA-3A, and EBNA-3B, or (d) PBS. Animals vaccinated with soluble gp350 produced higher levels of antibody to the glycoprotein than those vaccinated with VRPs expressing gp350. Animals vaccinated with VRPs expressing EBNA-3A and EBNA-3B developed LCV-specific CD4 and CD8 T cell immunity to these proteins, while VRPs expressing gp350 did not induce detectable T cell immunity to gp350. After challenge with rhesus LCV, animals vaccinated with soluble rhesus LCV gp350 had the best level of protection against infection based on seroconversion, viral DNA, and viral RNA in the blood after challenge. Surprisingly, animals vaccinated with gp350 that became infected had the lowest LCV DNA loads in the blood at 23 months after challenge. These studies indicate that gp350 is critical for both protection against infection with rhesus LCV and for reducing the viral load in animals that become infected after challenge. Our results suggest that additional trials with soluble EBV gp350 alone, or in combination with other EBV proteins, should be considered to reduce EBV infection or virus-associated malignancies in humans.

## Author Summary

Epstein-Barr virus (EBV) is the primary cause of infectious mononucleosis and is associated with several cancers. Presently there is no licensed vaccine to prevent EBV diseases. Two types of candidate vaccines are under development; one involves immunization with the major glycoprotein (gp350) on the outside of the virus, while the other involves vaccination with EBV proteins expressed during latency. We compared these two types of candidate vaccines in a rhesus monkey model of EBV and found that the gp350 vaccine induced better protection from infection. In addition, animals that received the rhesus EBV glycoprotein and became infected had a lower level of rhesus EBV DNA in the blood at 23 months after challenge than animals that received the rhesus EBV latency protein vaccine that subsequently were infected. Since levels of EBV DNA in the blood have been predictive for EBV lymphomas in transplant patients, the ability of rhesus EBV gp350 to reduce levels of rhesus EBV in the blood after infection suggests the EBV gp350 could have a role in reducing certain EBV-associated cancers. This is the first test of candidate vaccines in the rhesus monkey model of EBV and shows that this model should be useful in further evaluation of EBV vaccines.

## Introduction

Epstein-Barr virus (EBV) is a causative agent of infectious mononucleosis and is associated with a number of malignancies including lymphomas in immunocompromised persons, Hodgkin lymphoma, Burkitt lymphoma, and nasopharyngeal carcinoma. Currently no vaccine has been licensed to prevent EBV infection or disease.

Most attempts to generate an EBV vaccine have focused on glycoprotein 350 (gp350) as the immunogen. gp350 is the most abundant EBV glycoprotein in virions and on the surface of infected cells. gp350 binds to CD21, the EBV receptor on B cells. EBV gp350 is spliced to form gp220. gp350 is important for virus absorption to B cells and soluble gp350 can block EBV infection. Antibodies to gp350 neutralize virus in vitro [1]. EBV gp350 protects cottontop marmosets from B cell lymphomas when challenged with high titers of EBV [2]. Numerous studies have shown that gp350 purified from cells [3], [4], expressed as a recombinant protein [5], [6], or expressed from an adenovirus [7] or vaccinia vector [8] can protect marmosets from EBV lymphomas. Vaccinia virus expressing gp350 induced EBV neutralizing antibody in seronegative children and a showed a trend toward protection from EBV infection [9]. Vaccination of young adults with recombinant gp350 in alum/monophosphoryl lipid A induced EBV neutralizing antibodies and protected EBV seronegative volunteers from infectious mononucleosis, but not from EBV infection [10], [11].

While gp350 is important for protection from infectious mononucleosis, EBV proteins expressed during latency are thought to be critical for controlling latent infection. The EBV nuclear antigen 3 (EBNA-3) latency proteins are the primary targets of CD8 T cells in the blood of healthy EBV carriers [12]. The success of treating patients with EBV lymphoproliferative disease with infusions of EBV-specific T cells [13], [14], in which the EBNA-3 proteins represent the immunodominant epitopes, indicates the critical role of these viral proteins for protection from EBV disease. The importance of T cell responses to EBNA-3B was demonstrated in a patient who died from an EBV lymphoma after the tumor cells developed a large deletion in EBNA-3B which allowed the malignant cells to escape from EBV-specific cytotoxic T cells [15]. A peptide corresponding to EBNA-3A was used in a small vaccine trial in EBV-seronegative human volunteers [16].

Given the complexities and costs of EBV vaccine trials in humans, testing vaccines in animal models might allow more rapid comparison of candidate vaccines. Many animal studies using gp350 have been performed in cottontop tamarins, which have several limitations. These animals cannot be infected with EBV by the oral route, they do not develop a persistent infection similar to humans, and the animals do not express MHC class I A, B or C alleles [17] which have been associated with virus-specific cytotoxic T cells (CTLs). In contrast, rhesus lymphocryptovirus (LCV) is naturally endemic in rhesus monkeys and reproduces most, if not all, of the features of EBV in these animals [18]. Infection of monkeys with rhesus LCV results in lymphadenopathy, splenomegaly, and atypical lymphocytes in some animals, and animals shed the virus from the oropharynx [19]. Unlike infection of cottontop tamarins with EBV, rhesus monkeys can be infected orally with rhesus LCV and the animals develop a persistent infection similar to that which occurs in humans. When animals are immunosuppressed some develop B cell lymphomas that contain rhesus LCV [20]. Rhesus LCV has an ortholog for each of the EBV genes; conversely each EBV gene has an ortholog in rhesus LCV [21]. The rhesus LCV genes can complement their human EBV orthologs in nearly all activities; thus, rhesus LCV should be an excellent model for studying EBV pathogenesis.

While EBV gp350 has been shown to be protective against tumors in cottontop tamarins challenged with high titers of EBV and one study showed that gp350 reduced the incidence of infectious mononucleosis in humans, no vaccine studies have been performed using rhesus LCV in monkeys. Furthermore no studies have been reported involving a direct comparison of different EBV vaccines, including gp350 versus EBV latency proteins, in the same trial.

We compared three rhesus LCV vaccines- (a) recombinant soluble rhesus LCV gp350, (b) rhesus LCV gp350 expressed from replication-defective, single cycle, virus-like replicon particles (VRPs) derived from an attenuated strain of Venezuelan equine encephalitis (VEE), and (c) a combination of rhesus LCV gp350, EBNA-3A, and EBNA-3B each expressed in separate attenuated VRPs for their ability to protect rhesus monkeys against infection with rhesus LCV and to determine their long term effect on rhesus LCV DNA in the blood after challenge.

## Materials and Methods

### Ethics statement

These experiments were approved by the Animal Care and Use Committees of the National Institute of Allergy and Infectious Diseases and the University of California, Davis. The studies were carried out in strict accordance with the recommendations in the Guide for the Care and Use of Laboratory Animals of the National Institutes of Health.

### Animals

Rhesus macaques were reared separately from rhesus LCV seropositive animals beginning at birth and serologic testing indicated that all animals were seronegative for rhesus LCV. Six to 18 month old animals were housed in pairs during the vaccination period, and housed separately after challenge. Animals were vaccinated by inoculation in the triceps muscle, and challenged with rhesus LCV by inoculation of the back of the throat with virus in 1 ml of cell culture media using a needleless syringe.

### Viruses

Rhesus LCV was isolated from LCL8664 cells (American Type Culture Collection, Manassas, VA). The cells were derived from a rhesus monkey with a malignant lymphoma [22]. LCL8664 cells were transfected with a plasmid expressing EBV BZLF1 using electroporation as described previously [23], and after 5 days the cells were pelleted and virus was isolated as reported previously [24]. Rhesus LCV was titrated as previously described for human EBV [25]. Briefly, serial dilutions of virus were incubated with 1×10^5^ rhesus peripheral blood mononuclear cells (PBMCs) and the cells were plated into wells of a 96 well plate with 0.5 ug/ml of cyclosporine A. After 6 weeks the titer of virus was determined by the method of Reed and Muench [26].

Modified vaccinia Ankara (MVA) expressing rhesus LCV gp350 and green fluorescent protein (GFP) was constructed by cloning rhesus LCV gp350 into plasmid pLW44 [27]. This plasmid contains the GFP gene linked to the vaccinia virus p11 promoter to facilitate screening of recombinant MVA. Due to a vaccinia transcription termination signal [28] in the rhesus LCV gp350 gene (TTTTTGT, sequence position 1147 to 1153), the 5′ half of the gene (1-1,291) was amplified by PCR using primers 5′-TCCCCCCGGGAACAATGGAAGCGGCTTTTCTG-3′ and 5′-ATACGCGTCGACTCTTCGGGTTGTCTGGTTGGAGC-3′ (Xma I and Sal I sites are underlined), and the PCR product was digested with Xma I and Sal I and inserted into the corresponding restriction sites of plasmid pLW44. The T at nucleotide 1147 was changed to C (resulting in no change in the amino acid sequence of gp350) using the Quick Change Site-Directed Mutagenesis kit (Stratagene) and the resulting plasmid was referred to as pLWrhgp350-mA. After confirmation of the sequence, the 3′ half of the gene was amplified using primers 5′-TCCCCCCGGGGCAGCCACAAATGTCACCGCTGTT-3′ and 5′-ATACGCGTCGACCTAAACAGCGGTTTCAAATTC -3′ (Xma I and Sal I sites underlined). The resulting PCR product was cut with XmaI and Sal I and inserted into the corresponding site of pLW44 to obtain plasmid pLWrhgp350-B. pLWrhgp350-mA was cut with Not I and Sex AI and the 5′ end of rhesus LCV gp350 was inserted into the Not I and Sex AI sites of pLWgp350-B to yield plasmid pLWrhgp350GFP. DF-1 (a chicken embryo fibroblast cell line) or primary chicken fibroblasts (a gift from Linda Wyatt, NIH) were infected with 0.05 plaque forming units (pfu) of MVA per cell and 2 hours later the cells were transfected with plasmid pLWrhgp350GFP. Plaques expressing GFP and rhesus LCV gp350 were isolated by successive rounds of plaque purification by freeze-thawing cells containing GFP-positive plaques and plating at limiting dilutions. The resulting virus, named MVA-gp350GFP, was propagated in DF-1 cells. To obtain MVA expressing rhesus LCV without GFP, plasmid pLWrhgp350GFP was digested with Kpn I which removes the GFP gene and religated to yield pLWrhgp350. DF-1 cells were infected with MVA-gp350GFP and 2 hours later were transfected with pLWrhgp350. Plaques that did not express GFP were isolated by plaque purification and the resulting virus was named MVA-gp350.

### Plasmids

#### gp350 constructs

The extracellular domain of rhesus LCV gp350 (amino acids 1–737 [21]) was cloned by PCR amplification using DNA isolated from LCL8664 cells and primers rhgp350-Sal (5′ATACGCGTCGACAACAATGGAAGCGGCTTTTCTG -3′) which contains a Sal I site (underlined) and rhgp350-TM-BglRv (5′- GAAGATCTTAGCATGGAGAGATTGGAGCCCTC-3′) which contains a Bgl II site (underlined). To construct a plasmid expressing rhesus LCV gp350 in cell culture, the PCR product containing the extracellular domain of rhesus LV gp350 was digested with Sal I and Bgl II and used as part of a three fragment ligation along with the Bgl II-Not I fragment of human Fc (derived from pDC409 [29]) and the Sal I-Not I fragment of pDC409. The resulting plasmid, pDCrhgp350-Fc, expresses the extracellular domain of rhesus LCV gp350 fused to the Fc domain of human IgG.

To construct pSGrhgp350, the rhesus LCV gp350 gene was amplified from LCL8664 cell DNA by PCR using primers rhgp350-F-EcoR containing an EcoR I site (CCGGAATTCAACAATGGAAGCGGCTTTTCTG) and rhgp350-BglRv (5′- CGCAGATCTCTAAACAGCGGTTTCAAATTCATCATC-3′) containing a Bgl II site (underlined) and inserted into the corresponding sites of pSG5 vector (Stratagene). To obtain pCIrhgp350, pSGrhgp350 was digested with Bgl II, blunted using T4 DNA polymerase, cut with EcoR I and the gp350 gene was cloned into the EcoR I-Sma I sites of pCI (Promega).

To measure antibody to rhesus LCV gp350, a plasmid was constructed containing the viral gene linked to the Renilla luciferase gene. The extracellular domain of the rhesus LCV gp350 gene was amplified by PCR using primers rhgp350-F-EcoR (see above) and rhgp350-TMR (5′- AAAGAATTCTAGCATGGAGAGATTGGAGCCCTC -3′) with an EcoR I site (underlined). The PCR product was digested with EcoR I and cloned into the corresponding EcoR I site of pREN3S to generate plasmid pREN3rhgp350.

#### EBNA-3 constructs

Since Jiang et al. [30] reported that rhesus LCV EBNA 3A begins at nucleotide 73,492, while Rivailler et al. [31] stated that the protein starts at nucleotide 73,534, we produced constructs expressing the former termed rhEBNA3A1 (in which EBNA-3A would start at the first methionine) and the latter termed rhEBNA3A2 (in which EBNA-3A would start at the second methionine). Rhesus LCV EBNA-3A1 was amplified from cDNA (since EBNA-3A mRNA is spliced near its 5′end) after RNA had been isolated from LCL8664 cells and cloned into TA-cloning vector pCR2.1 using the TOPO TA cloning kit (Invitrogen) and primers rhEBNA3A-FSal (5′- ACCGTCGACAAAATGGAGGAAGAAAGGCCG-3′) or rhEBNA3A2-FSal (5′-ACCGTCGACAACATGGAAGAAGAGGAGGTTCCATCC-3′) containing a Sal I site (underlined) and rhEBNA3A-RPst (5′-ACCCTGCAGTTATTCCTCATTATCTGGGGGATC-3′) with a Pst I site (underlined). Due to mutations in the 3′ portion in the cDNA noted after cloning, rhEBNA-3A1 was also amplified from DNA obtained from LCL8664 cells using same primers described above. The PCR product was then cut with Sal I and Pst I and inserted into the corresponding site of pLW44. To construct EBNA-3A1 and EBNA-3A2 without an intron, a three-fragment ligation was performed using (a) a Sal I-Pfo I fragment from the 5′ end of the EBNA-3A cDNA, (b) a Pfo I-Pst I fragment of EBNA-3A DNA, and (c) a Sal I-Pst I fragment from pLW44. The resulting plasmids were termed pLWrhEBNA-3A1 and pLWrhEBNA-3A2.

To clone EBNA-3A1 and EBNA-3A2 into an SV40 expression vector, PCR was performed with primer rhEBNA3A-FBcl 5′-ACCTGATCAAAAATGGAGGAAGAAAGGCCG-3′ or rhEBNA3A2FBcl 5′-ACCTGATCAAACATGGAAGAAGAGGAGGTTCCATCC-3′ with a Bcl I site (underlined) and rhEBNA3A-RBcl 5′-ACCTGATCATTATTCCTCATTATCTGGGGGATC-3′ with a Bcl I site (underlined) using plasmid pLWrhEBNA-3A as a template. The PCR products were cut with Bcl I and cloned into the Bam HI site of pSG5 to obtain pSGrhEBNA3A1 and pSGrhEBNA3A2.

To clone EBNA-3A1 into a CMV expression vector, pLWrhEBNA3A was digested with Pst I, blunted with T4 DNA polymerase, digested with Sal I, and the fragment containing EBNA-3A was inserted into the Sal I-Sma I site of pCI to produce pCIrhEBNA3A.

Rhesus LCV EBNA-3B was amplified from LCL8664 cell DNA as two separate fragments using primer pairs rhEBNA3BF-Sal (5′- ACCGTCGACAAAATGAAGAAAGCTTGGCTCGGC-3′) and rhEBNA3B GCR-Pst (5′-ACCCTGCAGTGGGGCAGCTGATATGGGGCGGCTCGC-3′ to generate the 5′ end of EBNA-3B), and primer pairs rhEBNA3B GCF-Sal (5′- ACAGTCGACACCCCCCGGGCACATATACCCGCCA-3′) and rhEBNA3BR-Pst (5′- ACCCTGCAGTTAGAACTCCTCGTCCGATATTTC-3′) to generate the 3′ end of EBNA-3B (Sal I and Pst I sites underlined). The 5′ EBNA-3B PCR product was cloned using the Invitrogen Zero Blunt TOPO PCR Cloning kit to generate pBluntrhEBNA3B-5′ and the 3′ EBNA-3B PCR product was cloned using the Invitrogen TOPO TA PCR Cloning kit to generate pCRrhEBNA3B-3′. The spliced region of rhesus LCV EBNA3B was amplified from cDNA obtained from RNA of LCL8664 cells using primers rhEBNA3BF-Sal and rhEBNA3BspR-Pst (5′- AAACTGCAGTATGACGCACAGTCATGCAGAGCC-3′; Pst I site underlined) and cloned using the Invitrogen Zero Blunt TOPO PCR Cloning Kit to obtain pBluntrhEBNA3B-spA. To produce a full length spliced EBNA-3B gene, a 3 fragment ligation was performed using (a) the Sal I-Bsa BI fragment containing the 5′ spliced end of EBNA-3B derived from pBluntrhEBNA3B-spA, (b) the Bsa BI-Xma I fragment containing the middle portion of EBNA-3B derived from pBluntrhEBNA3B-5′, and (c) the large Sal I-Xma I fragment containing the 3′ end of EBNA-3B and the plasmid vector derived from pCRrhEBNA3B-3′. The resulting plasmid, pCRrhEBNA3B, contains the full length spliced form of rhesus LCV EBNA-3B.

To clone EBNA-3B into a SV40 expressing vector, EBNA-3B was removed from plasmid pCRrhEBNA3B by digestion with Sal I and Pst I, the ends were blunted using T4 DNA polymerase and the EBNA-3B gene was inserted into the Bam HI site of plasmid pSG5 (after blunting with the Klenow fragment of DNA polymerase I) to obtain pSGrhEBNA3B. To clone EBNA-3B into a CMV expression vector, EBNA-3B was removed from plasmid pCRrhEBNA3B by digestion with Sal I and Not I and inserted into the corresponding sites of pCI to obtain pCIrhEBNA3B.

To remove the RBP-Jκ binding sites from rhEBNA-3A1 we deleted codons 204–207 (TFAC) corresponding to nucleotides 610–621 from pSGrhEBNA3A1. To remove the RBP-Jκ binding sites from rhEBNA-3B, we deleted codons 208 to 211 (TLGC) corresponding to nucleotides 622–633 from pSGrhEBNA3B using the Quik Change site-directed Mutagenesis kit (Stratagene). The resulting plasmids deleted for the EBNA-3 RBP-Jκ binding sites, pSGrhEBNA3A1-del, pSGrhEBNA3A2-del, and pSGrhEBNA3B-del, were only used for cloning into the Venezuelan equine encephalitis vector (see below). All gp350, EBNA-3A, and EBNA-3B constructs obtained by PCR were sequenced.

### Recombinant proteins

To produce rhesus LCV gp350-Fc protein, CV-1/EBNA-1 cells (ATCC, Manassas, VA) grown in DMEM/F-12 medium (1∶1) with 10% fetal bovine serum, were transfected with plasmid pDCrhgp350-Fc using DEAE-Dextran. After transfection, the media was changed to DMEM/F12 medium with 0.5% low immunoglobulin G fetal bovine sera (HyClone, Logan, UT). One week after transfection, the media was collected, clarified by low speed centrifugation, and filtered through a 0.45 um filter. Recombinant rhesus LCV gp350-Fc was bound to protein A-Sepharose beads, eluted from the beads with 12.5 mM citric acid pH 2.2, and collected in tubes containing 500 mM HEPES, pH 9.0 to neutralize the citric acid.

To produce recombinant rhesus LCV EBNA-3A and EBNA-3B, Cos cells were transfected with plasmid pSGrhEBNA3A1 or pSGrhEBNA3B using Lipofectamine 2000 (Invitrogen). Two days after transfection, lysates were prepared from the cells and proteins were separated by polyacrylamide gel electrophoresis. Proteins were stained with Coomassie blue, and bands containing EBNA-3A and EBNA-3B were excised from the gel. EBNA-3 proteins were eluted from the gel overnight in PBS and concentrated using a Centricon YM-100 filter (Millipore).

### Antibodies

To produce antibody to rhesus LCV gp350, 2 rabbits were immunized with 150 ug of rhesus LCV gp350-Fc fusion protein in complete Freund's adjuvant (Animal Pharm Service Inc., Sausalito, CA). Animals were boosted with 100 ug of gp350-Fc in incomplete Freund's adjuvant on days 28, 42, and 86 after the first vaccination; 2 weeks after the last boost the rabbits were bled and sera were obtained.

To produce antibody to rhesus LCV EBNA-3A and EBNA-3B, mice were immunized three times, 3 weeks apart with 100 ug of pCIrhEBNA3A or pCIrhEBNA3B. Three weeks after the 3rd DNA immunization, the animals were boosted with 20 ug of EBNA-3A protein or 15 ug of EBNA-3B protein in complete Freund's adjuvant. Serum was collected from the mice 2 weeks later.

### Vaccines

For rhesus monkey vaccinations, rhesus LCV gp350-Fc protein was incubated with Alhydrogel 2% (Brenntag Biosector, Accurate Chemical and Scientific Corp) by mixing on a rotating wheel for 30 min at room temperature followed by addition of monophosphoryl lipid A (Avanti Polar Lipids, Inc., Alabaster, AL).

Replication-defective attenuated Venezuelan equine encephalitis viruses (VEE) expressing rhesus LCV gp350, EBNA-3A1, or EBNA-3B were constructed by PCR amplification of the genes from plasmids pSGrhgp350, pSGrhEBNA3A1-del, pSGrhEBNA3B-del and inserting the rhesus LCV genes into a VEE replicon vector. The replicon vector contains the VEE nsP1, nsP2, nsP3, and nsP4 genes and an internal ribosome entry site (IRES) followed by a cloning site into which the rhesus LCV genes were inserted [32]. RNAs were produced from the replicon and VEE plasmid vectors using T7 polymerase. Vero cells were co-transfected with (a) helper RNA expressing VEE capsid, (b) helper RNA expressing VEE glycoprotein [33], and (c) RNA obtained from replicon vectors expressing rhesus LCV gp350, EBNA-3A, or EBNA-3B. The resulting transfections generated replication-defective, single-cycle, virus-like replicon particles (VRPs) [32], [34].

### Antibody testing for rhesus LCV and for gp350

Antibody to rhesus LCV viral capsid antigen (VCA) was determined by immunofluorescence (VRL Laboratories, San Antonio, Texas).

Antibody to rhesus LCV gp350 was measured using the luciferase immunoprecipitation system (LIPS) assay [25]. Cos cells were transfected with pREN3rhgp350 which encodes a fusion protein containing the rhesus LCV gp350 gene linked to the Renilla luciferase gene. Activity in transfected Cos cell lysates was determined by luminometry and expressed as luminometer units (LU) per ml as described previously [25]. To measure rhesus LCV gp350 antibody levels in animals, rhesus monkey plasma were diluted 1∶10, and 1 ul was added to 1×10^7^ light units (LU) of transfected Cos cell extract. Immunoprecipitations were performed by addition of protein A/G beads, and LU were determined by luminometry. A cut-off threshold limit was derived from the mean value plus 2 standard deviations of the background LU. All LU data shown represent the average of two independent experiments.

### Real-time PCR to quantify rhesus LCV DNA and RNA

DNA was isolated from 1−5×10^6^ PBMCs using either a QIAamp DNA Blood Mini Kit (Qiagen) or an Easy-DNA Kit (Invitrogen). Real time PCR for rhesus LCV DNA was performed with primers and probes that amplify rhesus LCV EBER1 [35] using the following conditions: 94°C for 15 sec, 60°C for 30 sec, and 72°C for 35 sec for a total of 40 cycles. Real time PCR was also performed to amplify the internal repeat 1 (IR1) region of rhesus LCV (which corresponds to the EBV Bam HI W fragments) with primers 5′-AAATCTAAACTTTTGAGGCGATCTG-3′ and 5′-CCAACCATAGACCCGTTTCCT -3′ and probe 5′-(6-Fam)-TCTCCGCGTGCGCATAATGGC-(TAM RA)-3′ using the following conditions: 50°C for 2 min and 94°C for 10 min for 1 cycle, followed by 94°C for 15 sec, 60°C for 1 min, for 45 cycles. Viral DNA was normalized using GAPDH [31] and results were expressed as DNA copies per 1×10^6^ PBMCs.

Real time reverse-transcriptase PCR was performed for rhesus LCV EBER1. Total RNA was isolated from 5×10^6^ PBMCs using Trizol (Invitrogen, Calsbad, CA), and reverse transcription and PCR was performed using primers and probes as described previously [35] and PCR conditions described above.

### Rhesus LCV-specific CD8 and CD4 T cell responses

Rhesus monkey cell lines were used to present EBNA-3A, EBNA-3B, and gp350 to rhesus PBMCs. Lymphoblastoid cell lines (LCLs), which express EBNA-3A and EBNA-3B, were constructed for each monkey by infecting PBMCs with rhesus LCV in the presence of cyclosporine A (500 ng/mL, Sigma-Aldrich) and culturing the cells in RPMI 1640 with GlutaMax (Invitrogen) with 10% FBS and antibiotics. Cryopreserved PBMCs were thawed and cultured in RPMI 1640 with GlutaMax with 10% FBS, IL-2 (5 U/mL, from the National Cancer Institute) and antibiotics in 12-well plates overnight. The following day, PBMCs were divided into 2 tubes (1−3×10^6^ cells per tube) and were cocultured with 2×10^6^ autologous LCLs for 5 hours in the presence of 10 µg/mL of brefeldin A (Sigma-Aldrich) and 10 U/mL of IL-2. The cells were then washed in PBS with 2% FBS and 2 mM EDTA, incubated with FITC-conjugated anti-CD8 monoclonal antibody (clone RPA-T8, BioLegend, San Diego, CA) and APC-conjugated anti-CD4 monoclonal antibody (clone OKT4, BioLegend) for 20 min, washed with PBS containing 2% FBS and 2 mM EDTA, incubated with Cytofix/Cytoperm buffer (BD Bioscience, Franklin Lakes, NJ) for 25 min, and washed with Perm wash buffer (BD Bioscience). Cells were then incubated with PE-conjugated anti-IL-2 monoclonal antibody (clone MQ1-17H12, BioLegend) and PE-Cy7-conjugated anti-IFN-γ monoclonal antibody (clone B27, BD Bioscience), washed with Permwash buffer, and resuspended in PBS with 2% FBS and 2 mM EDTA. As a negative control, PBMCs were cultured without LCLs, and mixed with LCLs after fixation of PBMCs with Cytofix/Cytoperm buffer. Data were acquired using a FACS Caliber (BD Bioscience) and analysis was performed using Flowjo software 8.8.4 (Tree Star Inc., Ashland, OR). The percent of rhesus LCV-specific cytokine T cell response was defined as the percent cytokine (IL-2 or IFN-γ, or both) positive CD4 or CD8 cells in LCL-stimulated PBMCs minus the percent cytokine positive CD4 or CD8 cells in unstimulated PBMCs. If unstimulated samples had a higher frequency of cytokine positive cells than stimulated samples, a value of 0% was assigned, instead of a negative value.

To measure gp350-specific CD8 and CD4 T cell responses, LCLs were infected with either wild-type MVA, or MVA expressing rhesus LCV gp350, at 3 TCID_50_ for 24 hours before coculture with PBMCs. PBMCs were thawed and cultured overnight as described above. The following day, PBMCs were divided into 3 tubes (0.8−2×10^6^ cells per tube) and cocultured with LCLs infected with either wild-type or gp350 expressing MVA for 5 hours in the presence of brefeldin A. As a negative control, PBMCs were cultured without LCLs and mixed with LCLs (not infected with MVA) after fixation of PBMCs with Cytofix/Cytoperm buffer. Staining and flow cytometry were done as described above. The percent of cytokine producing CD4 or CD8 cells in unstimulated PBMCs mixed with LCLs (not infected with MVA) after fixation (negative control) was subtracted from the percent of CD4 or CD8 cells in PBMCs stimulated with gp350 MVA-infected LCLs or wild-type MVA-infected LCLs. The percent of rhesus LCV gp350-specific CD4 or CD8 T cell response was defined as the percent of cytokine producing CD4 or CD8 cells in PBMCs stimulated with gp350 MVA-infected LCLs minus the percent of cytokine producing CD4 or CD8 cells in PBMCs stimulated with wild-type MVA-infected LCLs.

## Results

### Antibody to rhesus LCV gp350 detects the glycoprotein in cells infected with MVA-gp350

**Figure 1:**
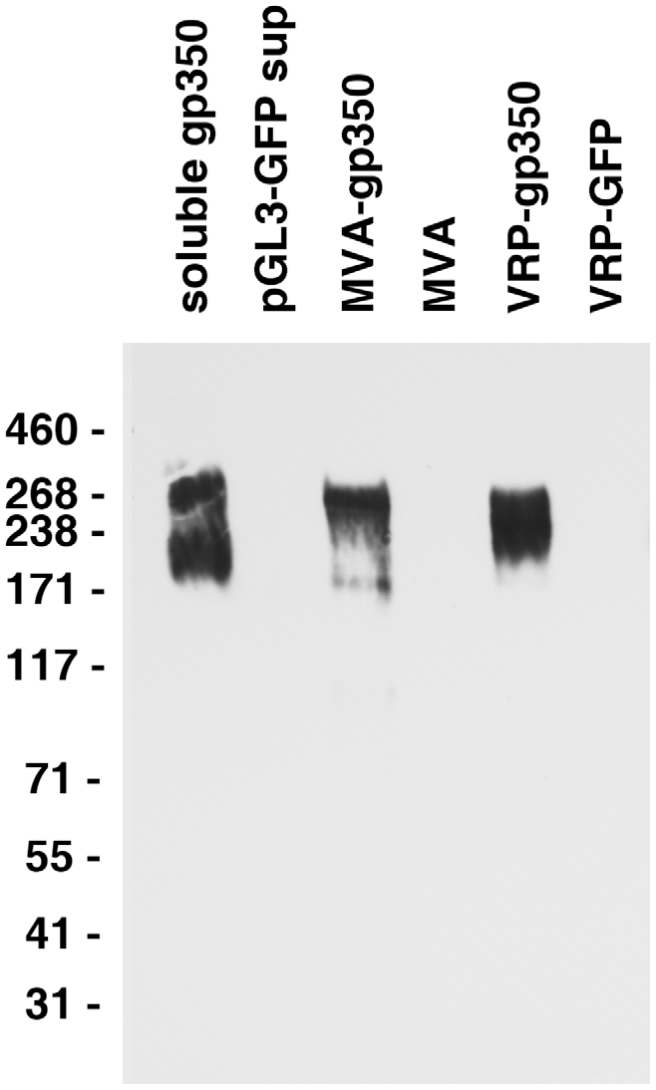
Detection of gp350 in supernatant from cells transfected with a plasmid expressing soluble gp350, lysate from DF-1 cells infected with MVA-gp350, and lysate from Vero cells infected with VRP-gp350. Supernatant from cells transfected with control plasmid pGL3-GFP, or lysates from cells infected with MVA or VRP-GFP are negative controls.

In order to determine if rhesus LCV encodes gp350 similar to its human EBV homolog, rabbits were immunized with purified rhesus LCV gp350-Fc fusion protein and serum was obtained. The rabbit serum detected proteins from 200−270 kDa in supernatant from cells transfected with plasmid expressing soluble gp350-Fc, but not with plasmid expressing GFP (pGL3-GFP) **(** **Fig. 1** **, lanes 1, 2)**.

To ensure that the rabbit antibody was specific for rhesus LCV gp350, we determined that the antibody could detect full length gp350 in virus-infected cells. Full length rhesus LCV gp350 was inserted into modified vaccinia Ankara (MVA). DF-1 cells were infected with MVA-gp350GFP or MVA alone and 16−24 hr later, lysates were prepared, and immunoblotted with the rabbit serum. Cells infected with MVA-gp350GFP, but not MVA alone produced a 250 kDa protein that reacted with the antibody **(** **Fig. 1** **, lanes 3, 4)**. Similarly, Cos cells infected with virus-like replicon particles expressing rhesus LCV gp350 (VRP-gp350), but not cells expressing GFP (VRP-GFP), expressed proteins of 220−250 kDa that reacted with the antibody (**Fig. 1** **, lanes 5, 6**). We were unable to detect rhesus LCV gp350 in LCL8664 cells treated with sodium butyrate or transfected with a plasmid expressing EBV BZLF1 (data not shown), likely due to low levels of the glycoprotein.

### Sequence and expression of rhesus LCV EBNA-3A and EBNA-3B

**Figure 2:**
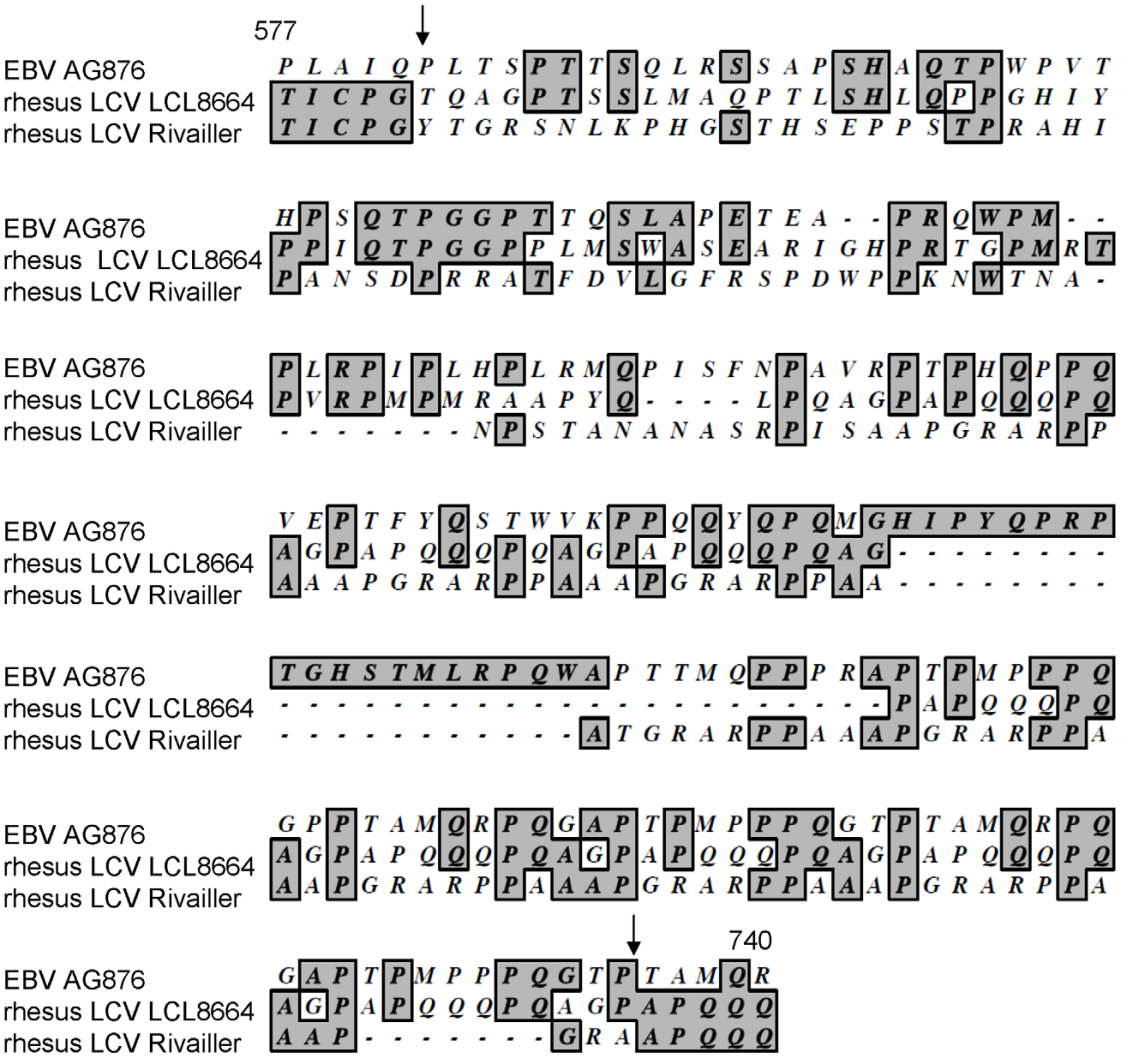
Alignment of amino acids 577−740 of rhesus LCV EBNA-3B from LCL8664 cells (middle lines) with the previously published sequence ([**21**], lower lines), and amino acids 571−768 of EBV AG876 EBNA-3B (top lines). Numbers indicate amino acid positions of rhesus LCV EBNA-3B and arrows indicate where the sequences of rhesus LCV EBNA-3B diverge and then return to identity.

In order to express rhesus LCV EBNA-3A and EBNA-3B, we cloned the genes from LCL8664 cells into expression vectors and determined the sequence of the viral genes. While the sequence of rhesus LCV EBNA-3A was identical to the published sequence [21], the sequence of rhesus LCV EBNA-3B was different. We found a T deleted at nucleotide 1744 and a C inserted at nucleotide 2206 of rhesus LCV EBNA-3B. The deletion at nucleotide 1744 results in a frameshift in the EBNA-3B sequence beginning at codon 582, and the insertion at nucleotide 2206 restores the open reading frame to the published amino acid sequence at codon 735 so that the last 193 amino acids of the protein are unchanged (**Fig. 2**). This was verified for several PCR clones from LCL8664 cells and by direct sequencing of DNA from LCL8664 cells. Comparison of the amino acid sequence of rhesus LCV EBNA-3B reported here, in the region just prior and after the frameshift mutation (acids 577−740), with that of EBV AG876 EBNA-3B showed 31% identity, while comparison of the prior rhesus LCV EBNA-3B sequence [21] with EBV AG876 EBNA-2B showed only 16% identity. Taken together these findings suggest that the sequence reported here for rhesus LCV EBNA-3B is more likely to be the authentic sequence of the protein.

Both EBV EBNA-3A [36] and EBNA-3B bind to RBP-Jκ and stimulate B cell proliferation. Since EBV EBNA-3A is critical for B cell growth transformation and survival [36] and EBNA-3-induced B cell proliferation might be problematic for a vaccine, we deleted the RBP-Jκ binding sites in rhesus LCV EBNA-3A and EBNA-3B. Mutation of the EBV EBNA-3A RBP-Jκ binding domain, TLGC (amino acids 199−202), to AAGA results in loss of function of the protein and reduces its ability to bind to RBP-Jκ. Rhesus LCV EBNA-3A and EBNA-3B also bind to RBP-Jκ [30]. Alignment of the amino acid sequence of rhesus LCV EBNA-3A with its EBV homolog predicts that the rhesus LCV EBNA-3A RBP-Jκ binding site TFAC (amino acids 204 to 207 based on the sequence of Jiang et al. [30], or amino acids 190−193 based on the sequence of Rivailler et al. [31]) are positional homologs of the RBP-Jκ binding site TLGC (amino acids 199−202) of EBV EBNA-3A. Similarly, alignment of rhesus LCV EBNA-3B with EBV EBNA-3B predicts that amino acids 208 to 211 (TLGC) of rhesus LCV EBNA-3B are positional homologs of EBV EBNA-3B amino acids 205 to 208 (TLGC). Therefore, we deleted these four codons from rhesus LCV EBNA-3A and EBNA-3B in vectors expressing these genes.

**Figure 3:**
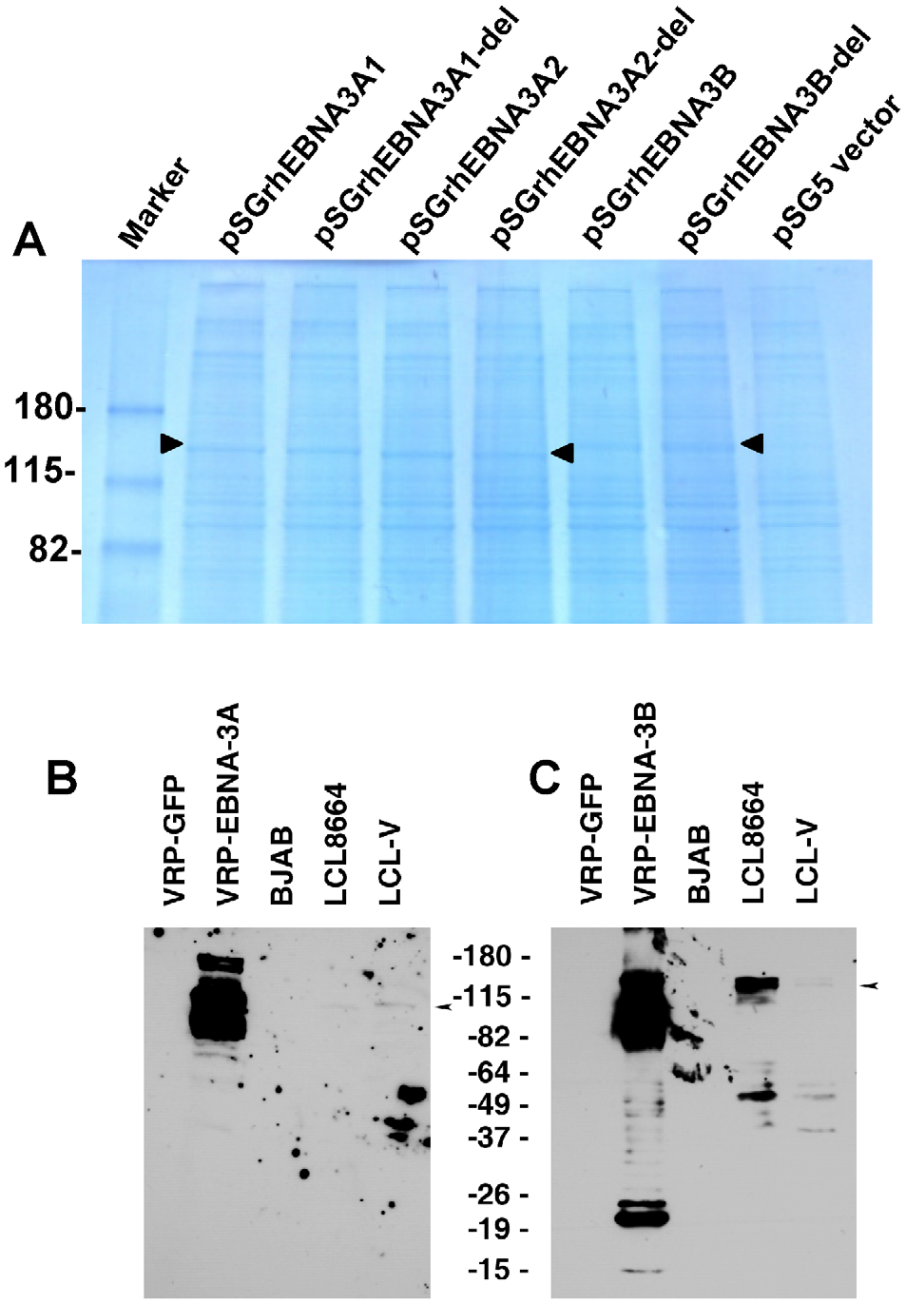
Expression of EBNA-3A and EBNA-3B in transfected and latently infected cells. (A) Cos cells transfected with pSGrhEBNA3A1 and pSGrhEBNA3A1-del express EBNA-3A beginning at the first methionine without (lane 2) or with (lane 3) a deletion in the putative RBP-Jκ binding site, respectively. Cos cells transfected with pSGrhEBNA3A2 and pSGrhEBNA3A2-del express EBNA-3A beginning at the second methionine without (lane 4) or with (lane 5) a deletion in the putative RBP-Jκ binding site, respectively. Cos cells were transfected with pSGrhEBNA3B and pSGrhEBNA3B-del, with a deletion in the putative RBP-Jκ binding site (lanes 6 and 7, respectively). Cos cells were transfected with empty vector (pSG5, lane 8). (B) Detection of EBNA-3A in Vero cells infected with VRP-EBNA-3A, or in LCL8664 cells and in a rhesus monkey LCL (LCL-V). Arrow indicates location of EBNA-3A. (C) Detection of EBNA-3B in Vero cells infected with VRP-EBNA-3B, or in LCL8664 cells and LCL-V. Arrow indicates location of EBNA-3B. Additional bands noted in cells infected with VRP expressing EBNA-3A or -3B are likely due to overexpression of the protein.

Based on the sequence of rhesus LCV EBNA3A (rhEBNA3A), either of two methionines could be the first amino acid of the protein [30]. To determine which can be used for EBNA-3A, we made four EBNA-3A constructs- rhEBNA-3A1 (which starts at the first methionine), rhEBNA-3A2 (which starts at the second methionine), rhEBNA-3A1-del and rhEBNA3A2-del (in which the four amino acid putative RBP-Jκ binding site in EBNA-3A was deleted). Transfection of Cos cells with pSGrhEBNA3A1, pSGrhEBNA3A2, pSGrhEBNA3A1-del, and rhEBNA3A2-del followed by Coomassie blue staining of cell lysates in PAGE gels showed bands of 147 kDa, 145 kDa, 147 kDa, and 144 kDa, respectively (**Fig. 3A** **, lanes 2−5**). These studies indicate that either the first or second methionine can be used for producing EBNA-3A. Transfection of Cos cells with pSGrhEBNA3B or pSGrhEBNA3B-del (deleted for the four amino acid putative RBJ-κ binding site) yielded a band of 150 kDa (**Fig. 3A** **, lanes 6,7**). Vero cells infected with VRP-EBNA-3A showed predominant bands of 102 and 88 kDa, while LCL8664 cells and rhesus LCV LCL-V showed a band of about 102 kDa (**Fig. 3B**). Vero cells infected with VRP-EBNA-3B showed an upper band of 145 kDa and more intense bands from 105−120 kDa while LCL8664 cells and rhesus LCV LCL-V showed a band of 145 kDa (**Fig. 3C**).

### Recombinant soluble gp350 elicits higher antibody titers to gp350 than VRP-gp350 in rhesus monkeys

Four rhesus LCV seronegative monkeys each received one of four inocula intramuscularly: (a) 50 ug of rhesus LCV soluble gp350-Fc protein (soluble gp350) formulated in 800 ug alum and 50 ug monophosphoryl lipid A, (b) 1×10^8^ infectious units (IU) of virus-like replication-defective VEE particles expressing rhesus LCV gp350 (VRP-gp350) in 1 ml of DMEM with 10% FBS, (c) a combination of three separate replication-defective VEE particles expressing rhesus LCV gp350 (VRP-gp350), EBNA-3A (VRP-EBNA-3A), and EBNA-3B (VRP-EBNA-3B) each at a titer of 1×10^8^ IU in a total of 1 ml of DMEM with 10% FBS, or (d) PBS control. The rhesus LCV soluble gp350 used in our vaccine contains the extracellular domain of the glycoprotein fused to the Fc domain of human IgG, while the vaccine used in the large human trial [11] has the extracellular domain of EBV gp350 with a mutation in the gp220 splice site and no Fc protein fused to the glycoprotein. The alum/monophosphoryl lipid A adjuvant was chosen for rhesus LCV soluble gp350, since this is the adjuvant that was used in the large human EBV gp350 study [11]. Animals were vaccinated at weeks 0, 4, and 12.

**Figure 4:**
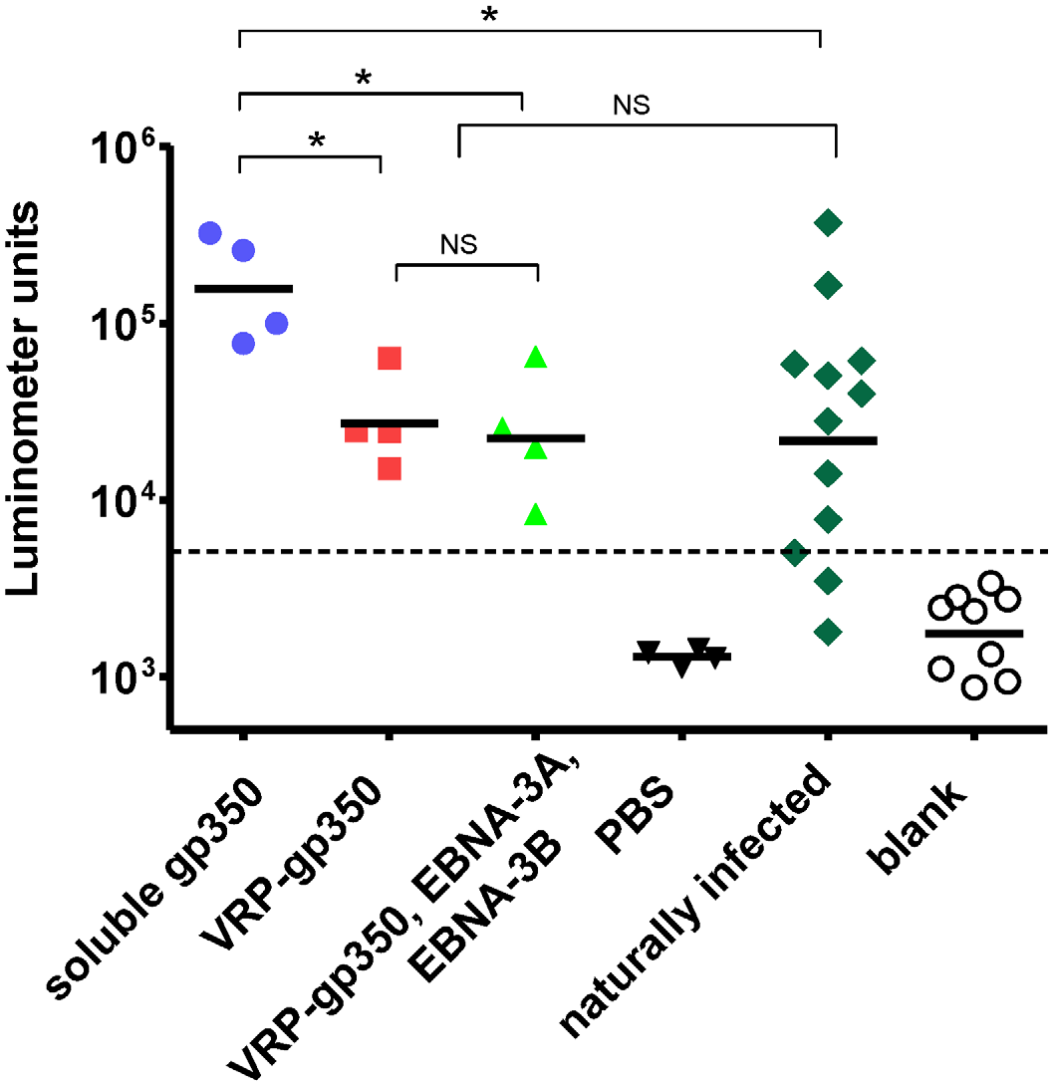
Detection of gp350 antibody by luciferase immunoprecipitation assay in rhesus monkeys immunized with soluble gp350, VRP-gp350, a combination of VRP-350, VRP-EBNA-3A, and VRP-EBNA-3B, or PBS before challenge with wild-type virus. gp350 antibody levels in naturally infected monkeys are shown. Antibody levels are measured as luminometer units. Cut off value is shown as horizontal dotted line, which was determined as the mean +2 standard deviations of the blank signal (open circles). Horizontal bars indicate geometric mean, asterisks indicate p<0.05 (Mann-Whitney's U-test), NS indicates not significant.

Serum antibody responses to gp350 in animals 5 weeks after the last vaccination showed that all animals vaccinated with soluble gp350 or VRP-gp350 (alone or in combination with VRPs expressing EBNA-3A and EBNA-3B) produced antibodies to the glycoprotein (**Fig. 4**). The geometric mean antibody level was significantly higher in animals vaccinated with recombinant soluble gp350 than in animals receiving VRP-gp350 (p<0.05) or in animals that had been naturally infected with rhesus LCV (p<0.05). The geometric mean antibody titer in animals receiving VRP-gp350 (alone or in combination with VRPs expressing EBNA-3A and EBNA-3B) was not significantly different than in animals naturally infected with rhesus LCV.

### Immunization of monkeys with VRPs expressing a combination of EBNA-3A, EBNA-3B, and gp350 induces rhesus LCV LCL-specific CD4 and CD8 T cell immune responses

**Figure 5:**
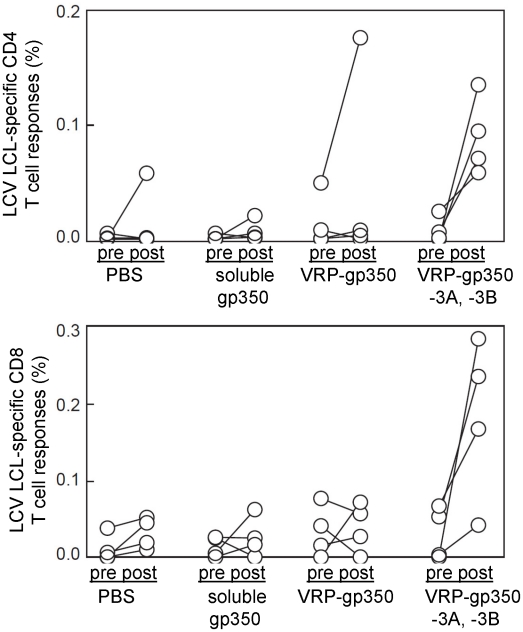
Detection of rhesus LCV-specific CD4 and CD8 T cell responses to gp350 or EBNA-3A and EBNA-3B in rhesus monkeys. Animals were immunized with soluble gp350, VRP-gp350, a combination of VRP-gp350, VRP-EBNA-3A, and VRP-EBNA-3B, or PBS before challenge with wild-type virus. Pre indicates PBMCs obtained before vaccination; post indicates PBMCs obtained after vaccination.

Rhesus LCV LCL-specific CD4 and CD8 T cell immune responses in monkeys were measured both pre- and post-vaccination. Rhesus LCV LCLs, which express EBNA-3A and EBNA-3B (**Fig. 3**), from each monkey served as antigen presenting cells. PBMCs from monkeys were incubated with autologous LCLs and cells were then stained for surface expression of CD4 and CD8 and for intracellular expression of IL-2 and IFN-γ. Before vaccination, the mean percentage of rhesus LCV-specific cytokine producing CD4 T cells was 0.0014±0.0013, 0.0013±0.0014, 0.0143±0.0135 and 0.0078±0.0045 (mean ± SE) for animals receiving PBS, soluble gp350, VRP-gp350, and combined VRP-gp350, VRP-EBNA-3A, and VRP-EBNA-3B, respectively **(** **Fig. 5** **)**. The mean percentage of rhesus LCV-specific CD4 T cells after vaccination was 0.0147±0.0165, 0.0070±0.0053, 0.0467±0.0500 and 0.0894±0.0516 for animals receiving PBS, soluble gp350, VRP-gp350, and combined VRP-gp350, VRP-EBNA-3A, and VRP-EBNA-3B, respectively. The mean percentage of rhesus LCV-specific cytokine producing CD8 T cells pre-vaccine was 0.0111±0.0105, 0.0140±0.0077, 0.0334±0.0194 and 0.0223±0.0178 for monkeys receiving PBS, soluble gp350, VRP-gp350, and combined VRP-gp350, VRP-EBNA-3A, and VRP-EBNA-3B, respectively. Post-vaccination, the mean percentage of rhesus LCV-specific CD8 T cells was 0.0315±0.0117, 0.0260±0.0152, 0.0390±0.0185 and 0.1834±0.1059 for PBS, soluble gp350, VRP-gp350, and combined VRP-gp350, VRP-EBNA-3A, and VRP-EBNA-3B, respectively. While there was considerable variability among individual animals, only animals receiving combined VRP-gp350, VRP-EBNA-3A, and VRP-EBNA-3B had a statistically significant increase in the percentage of rhesus LCV LCL-specific CD4 T cells (p = 0.032 by T-test). Animals that received combined VRP-gp350, VRP-EBNA-3A, and VRP-EBNA-3B had an increase in the percentage of rhesus LCV LCL-specific CD8 T cells after vaccination, but the difference did not reach statistical significance (p = 0.065 by T-test).

We did not observe CD4 or CD8 T cell responses to rhesus LCV gp350 in monkeys after vaccination using LCLs as antigen presenting cells, except for one animal that had an increase in rhesus LCV LCL-specific cytokine positive CD4 T cell response after vaccination with VRP-gp350 (**Fig. 5**). This is not surprising since we were unable to detect rhesus LCV gp350 in LCLs by immunoblot (data not shown). Therefore, we looked for T cells responses to rhesus LCV gp350 by infecting LCLs with either MVA-gp350 (which expresses rhesus LCV gp350) or wild-type MVA and used these LCLs as antigen presenting cells. We did not detect an increase in rhesus LCV gp350-specific PBMCs from animals vaccinated with soluble gp350 or VRP-gp350 (data not shown), even though MVA-gp350 expresses the glycoprotein (**Fig. 1**). It is possible that a different method to present gp350 to PBMCs would have been more effective; nonetheless, these data suggest that soluble gp350 or VRP-gp350 did not induce a significant increase in cellular immune responses in monkeys.

### Recombinant soluble gp350 protects against challenge from rhesus LCV better than VRP-gp350, or the combination of VRP-gp350, VRP-EBNA-3A, and VRP-EBNA-3B

A challenge inoculum of rhesus LCV was titered in LCV seronegative rhesus monkeys. Five animals were initially given 14 TID_50_ (infectious dose of virus needed to transform 50% of wells of cells in vitro) of rhesus LCV by application of virus to the throat and all animals seroconverted. Based on these results, 10 weeks after the last vaccination, animals were challenged by the oral route with 50 TID_50_ of rhesus LCV.

**Figure 6:**
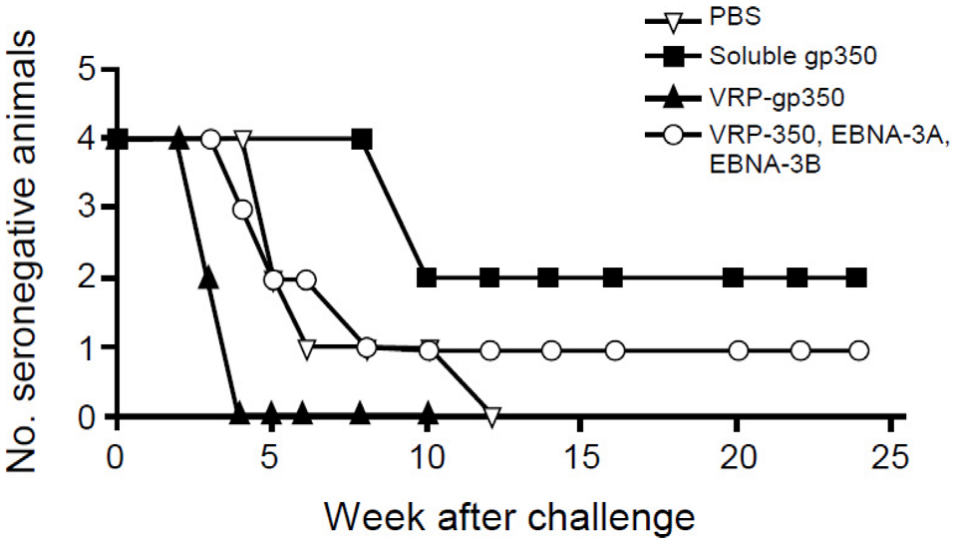
Detection of antibody to rhesus LCV VCA in monkeys immunized with soluble gp350, VRP-gp350, a combination of VRP-350, VRP-EBNA-3A, and VRP-EBNA-3B, or PBS.

Antibody to rhesus LCV viral capsid antigen (VCA) was detected after challenge with rhesus LCV in all 4 animals that received PBS and all 4 that received VRP-gp350. In contrast, 2 of 4 that received soluble gp350 and 3 of 4 animals that received a combination of VRP-gp350, VRP-EBNA-3A, and VRP-EBNA-3B developed antibody to rhesus LCV VCA after challenge (**Fig. 6**). Interestingly, seroconversion was delayed to week 10 after challenge in the 2 animals that received soluble gp350 that became infected, while seroconversion occurred in weeks 3 to 8 in most of the other vaccine groups.

**Figure 7:**
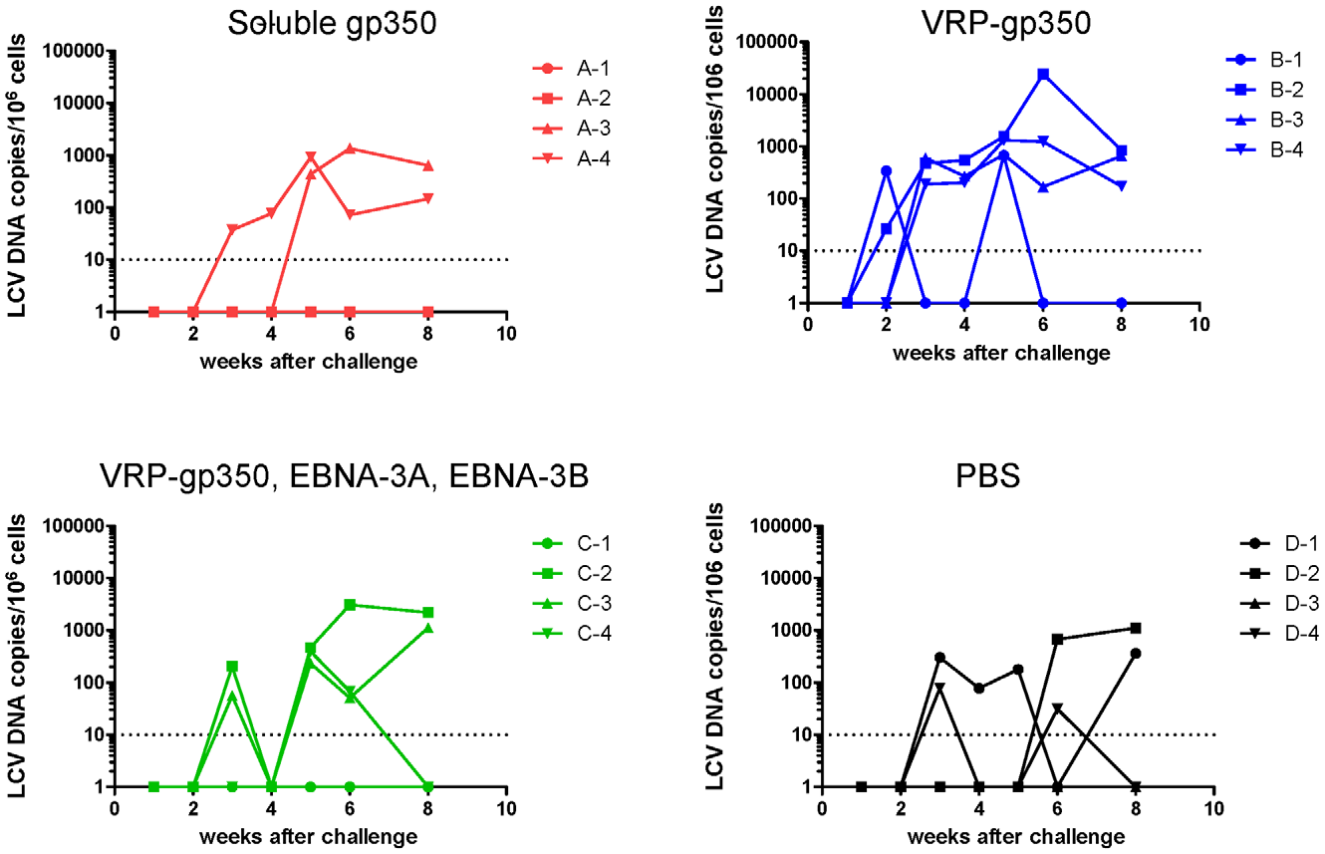
Detection of rhesus LCV DNA in the blood of monkeys immunized with soluble gp350, VRP-gp350, a combination of VRP-350, VRP-EBNA-3A, and VRP-EBNA-3B, or PBS. Real time PCR was performed using a probe that detects EBER1 DNA.

Like EBV, rhesus LCV DNA is present at very low or undetectable copy numbers in PBMCs of healthy animals infected in the past, but is usually detected in the blood after initial infection [31]. Within 6 weeks after challenge, rhesus LCV DNA was detected in PBMCs in 2 of 4 animals that received soluble gp350 and in 3 of 4 animals that received a combination of VRP-gp350, VRP-EBNA-3A, and VRP-EBNA-3B or PBS (**Fig. 7**). All 4 animals that received VRP-gp350 had detectable rhesus LCV DNA.

**Figure 8:**
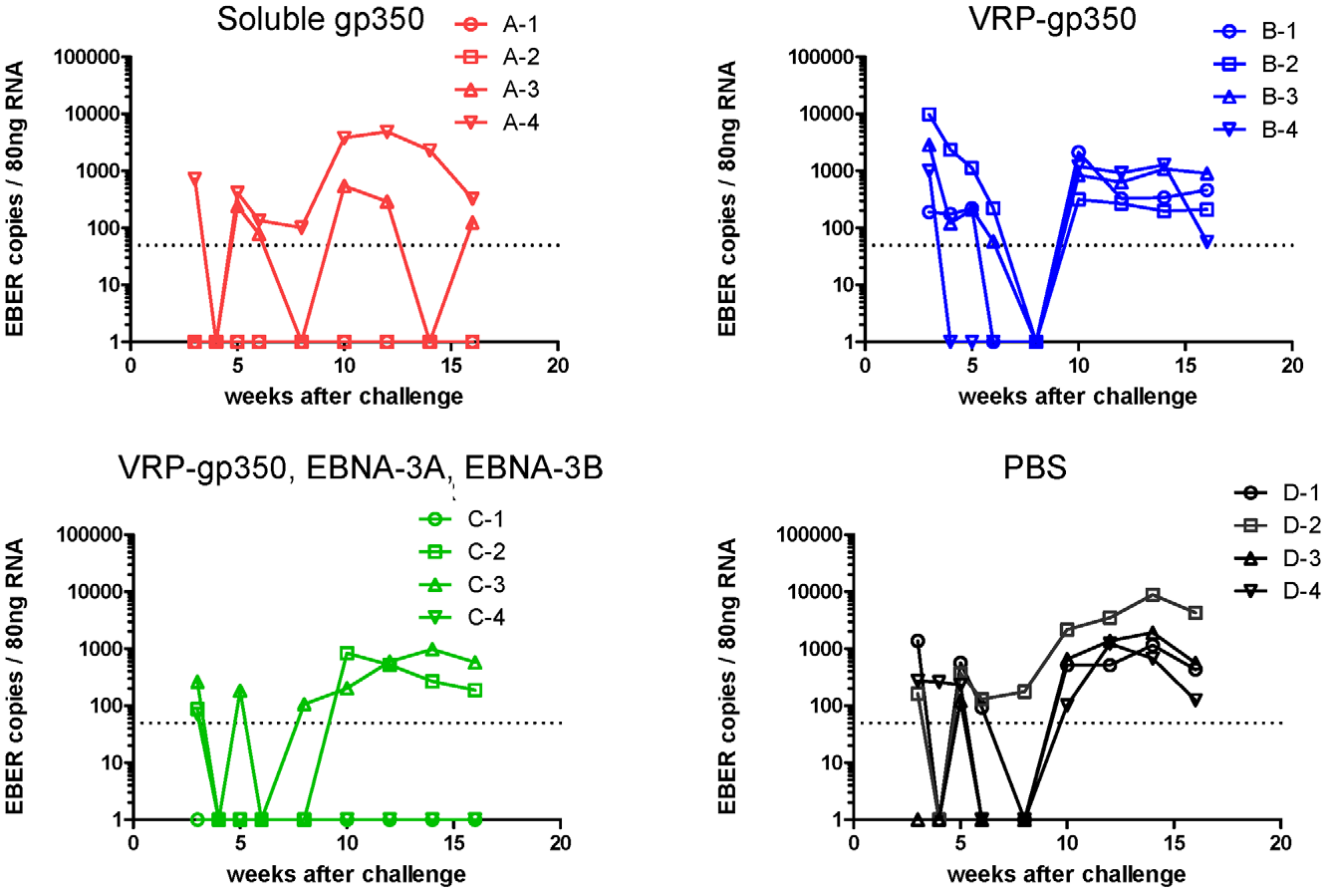
Detection of rhesus LCV EBER1 in the blood of monkeys immunized with soluble gp350, VRP-gp350, a combination of VRP-350, VRP-EBNA-3A, and VRP-EBNA-3B, or PBS. RNA was isolated from PBMCs and reverse transcription was performed followed by real time PCR with a probe that detects EBER1 DNA.

To further verify that animals were protected from infection after challenge we tested PBMCs from animals for rhesus LCV EBER1. EBER1 is present in thousands of copies in virus-infected B cells and is usually detected in the blood for life after infection of rhesus monkeys [35]. After challenge, rhesus LCV EBER1 was detected in PBMCs in 2 of 4 animals that received soluble gp350 and in 3 of 4 animals that received the combination of VRP-gp350, VRP-EBNA-3A, and VRP-EBNA-3B (**Fig. 8**). One animal that received the VRP-gp350, VRP-EBNA-3A, and VRP-EBNA-3B combination had a single positive level of EBER1 in the blood at 3 weeks after challenge and subsequently remained EBER1 negative. In contrast, all 4 animals that received VRP-gp350 or PBS had detectable EBER1 in the blood after challenge. Thus, all animals that seroconverted were positive for LCV EBER1.

In summary, animals receiving soluble gp350 had the best level of protection after challenge with the fewest numbers of animals with rhesus LCV DNA or rhesus LCV RNA in the blood and the lowest rate of seroconversion after challenge, while animals that received the combination of VRP-gp350, VRP-EBNA-3A, and VRP-EBNA-3B had the next best level of protection.

Analysis of fever, lymph node swelling, liver function tests, and CD4 to CD8 ratios after challenge did not show discernable differences in animals that received different vaccines or control PBS (data not shown). This may not be surprising since there were small numbers of animals, all animals were all <3 years old (and EBV infectious mononucleosis is rare in young children), and variability in animals after infection has been reported previously [19].

### Animals vaccinated with recombinant soluble gp350 have the lowest rhesus LCV loads at 23 months after infection with rhesus LCV compared with the other vaccines, and comparable viral loads to those vaccinated with the combination of VRP-gp350, VRP-EBNA-3A, and VRP-EBNA-3B at 34 months

**Figure 9:**
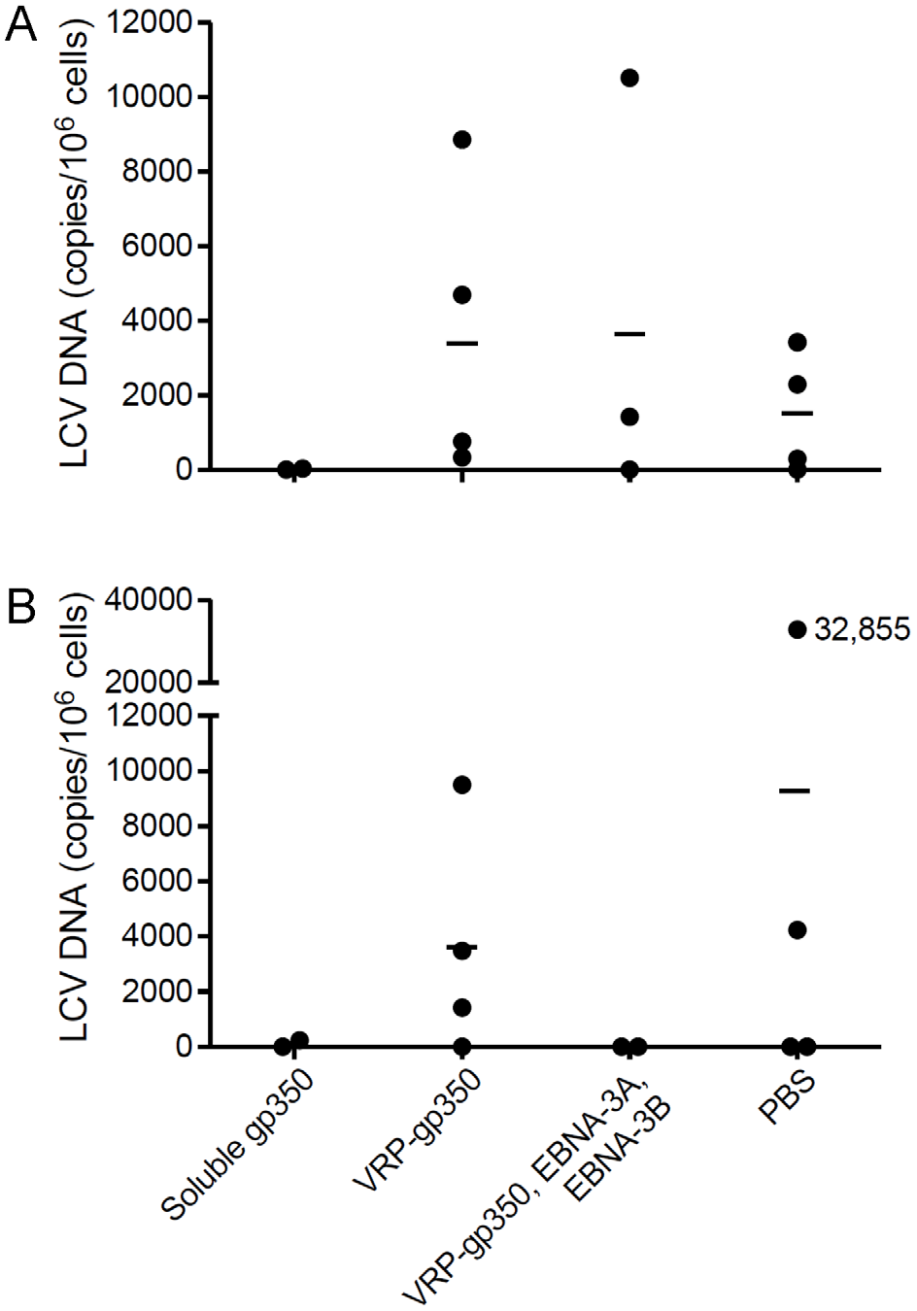
Detection of rhesus LCV DNA in the blood of monkeys immunized with soluble gp350, VRP-gp350, a combination of VRP-350, VRP-EBNA-3A, and VRP-EBNA-3B, or PBS and challenged with rhesus LCV. Blood was obtained 23 (A) and 34 months (B) after challenge. Real time PCR was performed using a probe that detects rhesus LCV IR1 (corresponding to the EBV Bam HI W repeat) DNA. Horizontal lines indicate mean values.

PBMCs were obtained from each of the vaccinated animals at 23 and 34 months after challenge. Using real time PCR with the rhesus LCV DNA EBER1 probe, we were only able to detect rhesus LCV DNA in PBMCs from 1 of the 16 animals 23 months after challenge (data not shown). Therefore, we developed a more sensitive real time PCR assay using IR1 DNA (corresponding to the Bam HI W repeats of EBV) that are present at 5.7 copies in the rhesus LCV genome [21]. Using the more sensitive real time PCR assay we were able to detect rhesus LCV DNA in 5 of 5 monkeys that had been naturally infected (data not shown). As expected we were unable to detect rhesus LCV DNA in animals that had been protected from challenge, therefore those animals were excluded from further analyses to avoid skewing the results. At 23 months after challenge, using the more sensitive real time PCR test, the mean rhesus LCV DNA copy number was 15 copies per 10^6^ cells for animals vaccinated with soluble gp350, 3,986 for animals that received the combination of VRP-gp350, VRP-EBNA-3A, and VRP-EBNA-3B, 3,663 for animals receiving VRP-gp350, and 1,504 for animals that received PBS (**Fig. 9A** **).** At 34 months after challenge, the mean rhesus LCV DNA copy number was 120 copies per 10^6^ cells for animals vaccinated with soluble gp350, 0 for animals that received the combination of VRP-gp350, VRP-EBNA-3A, and VRP-EBNA-3B, 3,605 for animals receiving VRP-gp350, and 9,271 for animals that received PBS (**Fig. 9B**). Thus, the rhesus LCV DNA copy number was lowest in animals vaccinated with soluble gp350 at 23 months after challenge, and was similar to the copy number in animals that received the combination of VRP-gp350, VRP-EBNA-3A, and VRP-EBNA-3B at 34 months. Analysis of seropositive animals showed that rhesus LCV DNA was detected 23 or 34 months after challenge in 1 of 2 animals that received soluble gp350, 2 of 3 animals that received the combination of VRP-gp350, VRP-EBNA-3A, and VRP-EBNA-3B, 4 of 4 animals that received VRP-gp350, and 3 of 4 animals that received PBS.

## Discussion

Two different types of vaccines have been developed to prevent disease and limit primary infection with EBV. Soluble EBV gp350 reduced the rate of infectious mononucleosis by 78% in young adults [11]. Alternatively, induction of cellular immunity to EBNA-3 has been proposed to limit the events occurring immediately after primary infection including virus replication in the throat and the expansion of virus-infected B cells [37]. Prior studies have shown that EBNA-3 epitopes are primary targets for EBV-specific CTLs in healthy persons, and therefore an EBV vaccine containing EBNA-3 epitopes has been proposed [38], [39]. A peptide corresponding to EBNA-3A elicited peptide-specific T cell responses in EBV-seronegative human volunteers; 4 of 4 seronegative volunteers seroconverted to EBV asymptomatically, while 1 of 2 placebo recipients infected with EBV developed infectious mononucleosis [16]. Since rhesus LCV is considered one of the best animal models for EBV infection, we compared rhesus LCV soluble gp350 with VRPs expressing gp350 or VRPs expressing a combination of gp350, EBNA-3A and EBNA-3B. Animals received three doses of the vaccines at 0, 4, and 12 weeks. We found that rhesus LCV soluble gp350 induced better protection against challenge virus than VRPs expressing a combination of gp350, EBNA-3A and EBNA-3B.

Animals vaccinated with soluble gp350 produced the highest levels of antibody to the glycoprotein and these levels were higher than those seen in monkeys naturally infected with rhesus LCV. Prior studies have shown that antibody to gp350 is likely the predominant component of neutralizing antibody to EBV [40], [41], [42]. In addition gp350 induces antibody-dependent cellular cytotoxicity which may also be important in controlling EBV infection [43]. Animals vaccinated with VRP expressing gp350, or VRPs expressing gp350, EBNA-3A, and EBNA-3B developed lower levels of antibody to gp350 and had less protection against acute infection than animals that received soluble gp350. Thus, the high levels of antibody to gp350 are likely important for protection against acute infection with rhesus LCV.

We compared soluble rhesus LCV gp350 with VRPs expressing gp350 with the expectation that expression of the viral glycoprotein in cells infected with VRPs might enhance the immunogenicity of gp350 beyond its ability to induce antibody. Animal studies have shown that neutralizing antibody to gp350 alone does not always correlate with protection from disease. When cottontop tamarins were vaccinated with replication-defective adenovirus expressing gp350, non-neutralizing antibody to gp350 was induced, but the animals were protected against lymphoma [7]. In contrast, when cottontop tamarins were vaccinated with gp350 in liposomes, high titers of neutralizing antibodies were induced, but the animals were not always protected from lymphoma [44]. These studies showed protection from development of lymphoma, rather than protection from infection. Immunization of common marmosets with gp350 in alum resulted in neutralizing antibodies in some animals, but protection from infection (defined by absence of seroconversion after challenge) did not correlate with the presence of neutralizing antibodies [45]. Somewhat surprisingly we found that rhesus LCV soluble gp350 induced better protection against challenge virus than VRP expressing gp350. Animals vaccinated with VRP expressing gp350 had antibody to the glycoprotein at levels comparable to animals naturally infected with rhesus LCV; however, the levels were significantly lower than in animals vaccinated with soluble gp350.

Animals vaccinated with alphavirus VRPs expressing EBNA-3A and EBNA-3B developed CD4 and CD8 cell responses to these proteins, while those vaccinated with VRPs expressing gp350 did not have detectable cellular responses to the glycoprotein. It is possible that the different methods used to present these antigens (LCLs naturally expressing EBNA-3A and EBNA-3B versus cells infected with MVA expressing gp350) could be responsible for these differences. Alphavirus VRPs target dendritic cells, which are highly efficient antigen presenting cells, and are effective for inducing cellular immunity [46]. Prior studies in humans show that EBV EBNA-3A, EBNA-3B, and EBNA-3C are the main targets of CD8 T cells in humans, while EBV EBNA-1 is the principal target of CD4 T cells (reviewed in [12]). While EBV gp350-specific CD8 T cells have been detected in patients during infectious mononucleosis [47] and gp350-specific CD4 T cells have been detected in healthy EBV carriers [48], [49], the level of these T cells has not been quantified relative to those against EBNA-3. In general, the level of T responses to structural proteins is generally lower than that to latent proteins in healthy EBV carriers (reviewed in [12]).

After challenge of animals with rhesus LCV, animals vaccinated with soluble rhesus LCV gp350 had the best level of protection based on levels of rhesus LCV DNA or RNA in the blood and lower rates of seroconversion. While animals that received VRP-gp350, VRP-EBNA-3A, and VRP-EBNA-3B had the next best level of protection from challenge and might have better protection from reactivation, than those receiving the other vaccine candidates, we could not test for protection against reactivation with the small number of animals in the current study. Although soluble gp350 induced the highest levels of antibody to gp350 and the best protection from acute infection, addition of potent EBV-specific T cell responses in combination with high levels of antibody might enhance the effectiveness of an EBV vaccine.

Although an ideal vaccine would protect from infection with EBV, a vaccine that reduces the EBV DNA load might also be useful. The EBV DNA load is a predictor for development of certain EBV-associated malignancies [50]. EBV DNA is increased in the blood of transplant recipients prior to the development of EBV post-transplant lymphoproliferative disease [51], and rituximab which lowers the viral load in the blood likely reduces the rate of post-transplant lymphoproliferative disease [52]. Patients with primary EBV infection after transplantation have high viral loads, and a 24-fold increased risk of post-transplant lymphoproliferative disease compared with seropositive transplant recipients [53]. Similarly, patients with HIV who progressed to B cell lymphoma had elevated levels of EBV in PBMCs and the level increased several months before developing lymphoma [54]. In order to determine if our vaccine reduced the level of rhesus LCV DNA in the blood, we developed a more sensitive assay for detection of viral DNA. With this assay we found that animals vaccinated with soluble gp350 that became infected with rhesus LCV after challenge had lower levels of rhesus LCV DNA in PBMCs at 23 and 34 months compared with animals that received vaccine control (PBS). Taken together these finding suggest that an EBV vaccine that reduces the viral load after infection might also reduce the risk for development of certain EBV-associated malignancies.

In summary, our findings indicate that a subunit vaccine that induces primarily humoral, rather than cellular immunity can result in a low virus load in animals that develop breakthrough infection after challenge with wild-type virus. At 23 months after challenge, animals vaccinated with soluble gp350 that became infected with rhesus LCV had ≥100-fold lower levels of rhesus LCV DNA in PBMCs than those vaccinated with VRP-gp350, or the combination of VRP-gp350, VRP-EBNA-3A, and VRP-EBNA-3B. Rhesus LCV DNA was still lower in PBMCs from animals vaccinated with soluble gp350 at 34 months after challenge compared with animals that received PBS. Thus, antibodies to a viral glycoprotein before challenge likely alter the primary infection in such a way as to result in a lower viral load years later. While the largest EBV subunit vaccine study performed to date showed that soluble gp350 protected against infectious mononucleosis, breakthrough infection still occurred; however, the authors did not report on the level of EBV DNA in the blood after breakthrough infection [11]. Based on our data, as well as observations of EBV DNA in PBMCs in certain malignancies, future EBV vaccine studies should test the ability of the vaccine to reduce viral loads in persons that become infected.
